# 

**Published:** 1856-05

**Authors:** 


*Last Number of the Volume.—To Subscribers.*—With this number we send bills to all those indebted to us, which we trust will draw forth a prompt response in the shape of a remittance. Our circumstances are such as to justify an appeal to all indebted to us to meet their obligations immediately.

During the past year the support accorded to the Journal has been such as to meet our most sanguine expectations. A rapidly increasing subscription list cheers us on, and, while our expenses are increasingly large, we need only the prompt support of our present list of subscribers to make this one of the most prosperous Journals in the country. Shall we not have it?

We call attention to our advanced terms. Those who wish to save 20 per cent. on the cost of the Journal, will take advantage of them. The increased rate will be strictly enforced against all not remitting at the beginning of the year.

We shall publish a large edition of the June No., in anticipation of the usual increase in our subscription list at this season, and to avoid the trouble we experienced last year from an inability to supply all our new friends with the June and July numbers.

To the many correspondents who have done so much to make our pages interesting during the year, we tender our warmest thanks. We are vain enough to believe that no medical periodical in the country has a more talented corps of contributors.

*Nelson's Northern Lancet.*—We are pleased to notice that this interesting periodical has bestowed an abusive article on us. While we are, perhaps, more than ordinarily sensitive to disparaging remarks from our brethren of the corps editorial, we care nothing for the opinion of the Lancet, never having ranked it as one of our confreres.

---

*American Medical Association.*—The coming meeting of this body at Detroit, promises to be a full one, and as interesting as previous occasions. The "Peninsular," for April, indicates an active preparation on the part of the profession of Michigan. The delegation from this region promises to be large.

